# Group concept mapping to develop a salon-based HPV self-collection intervention

**DOI:** 10.1007/s10552-025-02056-6

**Published:** 2025-08-27

**Authors:** Kelly N. B. Palmer, Namoonga M. Mantina, Adebola Adegboyega, Itunu O. Sokale, Kathleen Pryor, Azaria Suero-Davis, Trevon Brooks, Jennifer Hatcher

**Affiliations:** 1 Department of Health Promotion Sciences, University of Arizona College of Public Health, Tucson, AZ USA; 2 Present Address: Division of General Internal Medicine and Population Science, Department of Medicine, Heersink School of Medicine, University of Alabama at Birmingham, Birmingham, AL USA; 3 University of Kentucky College of Nursing, Lexington, KY USA; 4 Department of Medicine, Baylor College of Medicine, Houston, TX USA; 5 School of Nutritional Sciences and Wellness, University of Arizona College of Agriculture, Life & Environmental Sciences, Tucson, AZ USA; 6 University of Arizona College of Medicine, Phoenix, AZ USA; 7 Office of Diversity and Inclusion, University of Arizona, Tucson, AZ USA

**Keywords:** Cervical cancer screening, Community-based screening, Community engaged research, Mixed-methods, Health equity, Implementation science

## Abstract

**Background:**

Black women in the US face higher cervical cancer mortality rates due to delayed diagnoses linked to lower screening rates. Contributing factors include provider bias, costs, and limited access, particularly affecting women aged 40–64. While innovative approaches like clinical and home-based HPV self-collection exist, equitable dissemination remains challenging. Distributing HPV self-collection kits in unconventional sites like hair salons may offer a solution. Using concept mapping, we gathered community insights to design a salon-based cervical cancer screening intervention.

**Methods:**

We employed groupwisdom™ and REDCap platforms for community-based participatory intervention development. Participants included members of the Black Community Advisory Council of Tucson (Black CACTus), comprising hairstylists (*n* = 3), salon clients (*n* = 4), and healthcare providers(*n* = 3), all identifying as Black women aged 23–53. Concept mapping included: 1) Brainstorming statements, 2) Sorting statements into thematic clusters, 3) Rating importance and feasibility, 4) Reviewing the cluster map, and 5) Evaluating a draft intervention.

**Results:**

Brainstorming produced 39 statements, forming six clusters: 1) Program Promotion, 2) Insurance, Cost & Benefits, 3) Communication Considerations, 4) Information and Education, 5) Overall Logistics and Process, and 6) Sample Collection. Communication Considerations and Sample Collection were rated most important, with Communication Considerations also ranking highest for feasibility. Communication Considerations, Information and Education, and Sample Collection were the highest rated when importance and feasibility were considered together.

**Conclusions:**

Engaging community perspectives is essential for adapting cancer screening from clinical settings to community spaces like hair salons. This collaborative concept mapping approach identified strategies to enhance cervical cancer screening access and uptake among Black women.

## Introduction

Black women bear a disproportionate burden of cervical cancer, with significantly higher incidence rates compared to non-Hispanic White women (9.5 versus 7.0 per 100,000 women). Additionally, Black women are more than twice as likely to die from cervical cancer compared to White women and exhibit the highest mortality rates from this disease among all racial-ethnic groups in the United States (US) [1, 2]. The overall 5-year relative cervical cancer survival rate among Black women is 56%, compared to 69% for non-Hispanic White women, partly because Black women are more likely to be diagnosed with advanced-stage disease and poorly differentiated tumors. This inequality partly stems from deficiencies in screening history, leading to delayed diagnoses [3, 4]. Screening rates among Black women are notably lower than those among non-Hispanic White women, despite existing guidelines and recommendations for cancer screening. Various barriers contribute to this, including biased healthcare practices, financial constraints, and a lack of social support [5–7]. Given the increased risk for cervical cancer among this vulnerable population, researchers and public health workers must intervene to reduce the barriers to screening.

The collection of vaginal samples for human papillomavirus (HPV) testing by women themselves—a process known as self-sampling or self-collection—has widely been used in global areas where traditional clinical-based cervical cancer screening is challenging [8]. Studies have shown that self-collected vaginal specimens are as reliable, sensitive, and accurate as clinician-collected samples [9–11]. Self-collection for cervical cancer screening has been reported to be highly acceptable among female populations [12] and has improved cervical cancer screening participation [12, 13]. The World Health Organization (WHO) recommends the inclusion of HPV self-collection for cervical cancer screening, and 17 countries have self-collection incorporated in their national HPV screening programs [8, 14]. While the FDA recently approved self-collection for HPV testing, this is limited to clinic-based screening. While this advancement has the potential to increase cervical cancer screening engagement, it fails to address medical mistrust and low healthcare utilization by Black women.

One potential strategy to address cervical cancer screening disparities among Black women is the incorporation of self-collection cervical cancer screening initiatives in unconventional venues, such as hair salons. Hair care establishments are integral fixtures within Black communities and are widely accessible across the geographical landscape of the US, thereby offering increased opportunities to raise awareness and facilitate cervical cancer screening. Additionally, hairstylists play a significant social role in the lives of Black women and possess the ability to inspire their clients to engage in desired health behaviors, including cancer screening. It is well-documented that Black women trust and respect their hairstylists [15, 16]. While studies utilizing hairstylists to convey health information regarding chronic disease screening and other health practices have shown promise, there remains a need to develop strategies that bolster implementation and sustainability. Comprehending contextual factors is essential for transitioning from traditional settings (such as healthcare facilities) to community locales for cancer screening. Delving into these contexts is particularly crucial for encouraging the adoption and endurance of cancer screening initiatives within hair salons. Previous research on general health promotion in beauty salons revealed that both Black women and hairstylists regard the salon as an environment conducive to information exchange and social support, serving as a culturally "safe" space for Black women [17]. Black women also expressed a preference for their hairstylists to be trained in intervention content, model desired behavior, and collaborate with healthcare professionals in intervention endeavors [15]. The accessible, supportive (social and cultural) environment of the beauty shop makes it a viable setting for cervical cancer screening intervention. To this end, the objective of this study is to develop a culturally and contextually appropriate cervical cancer self-screening intervention for Black women to be delivered in hair salons.

## Methods

### Theoretical framework

The settings approach theory serves as the theoretical framework guiding this project. It is a Community-Based Participatory Research approach that engages multiple investors in cooperative learning while considering the physical, social, and cultural environment of the setting [18, 19]. A tenet of this theory is the contextualization of the determinants of a setting to be modifiable rather than the setting simply being a place to “host” an intervention. This provides the opportunity to examine the infrastructure of a setting and develop strategies for intervention implementation that are place-specific. Employing the settings approach theory facilitated an understanding of the hair salon’s unique culture and context in the development of the HPV self-sampling intervention.

### Group concept mapping and participants

**Fig. 1:**
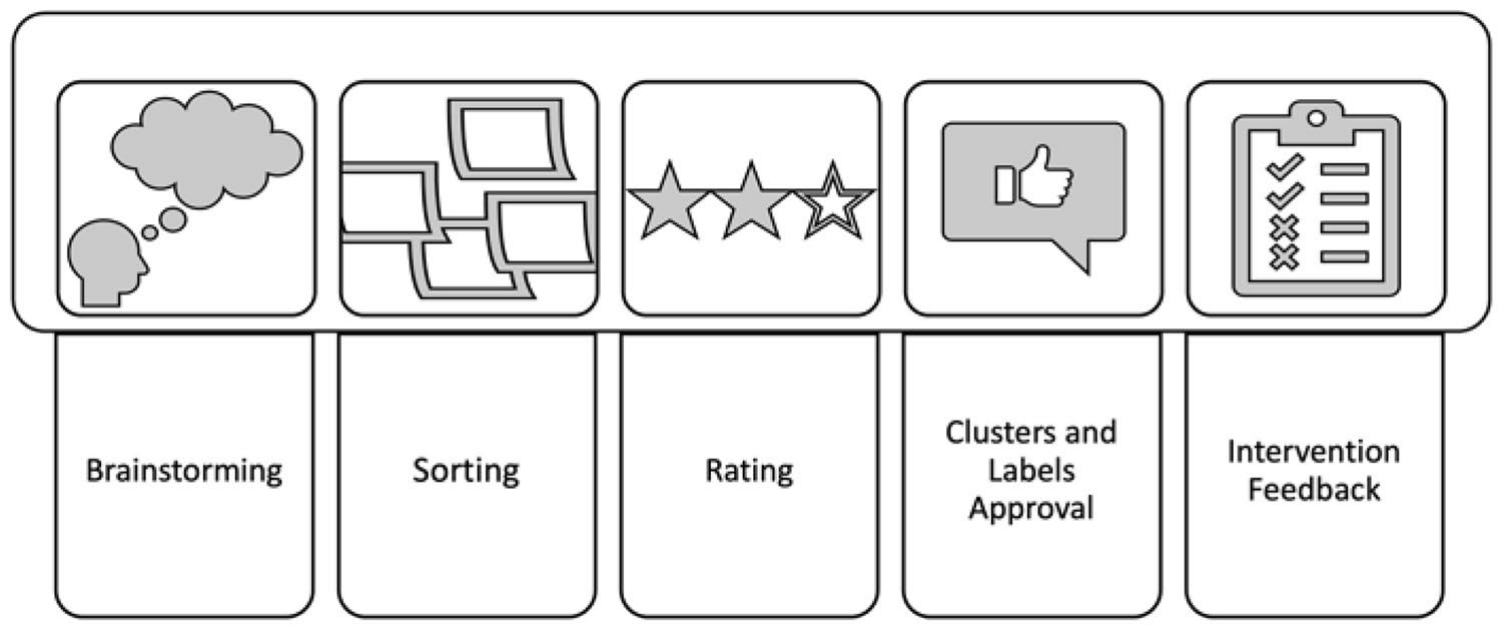
Group concept mapping process

The intervention was developed in a collaborative and participatory manner with our Black Community Advisory Council of Tucson (Black CACTus) using Group Concept Mapping (GCM), a mixed-methods approach. Group Concept Mapping integrates qualitative data collection methods with quantitative data analysis. Data are represented visually across a series of conceptually interconnected maps. Group Concept Mapping aligns with the settings approach theory in that it allows collaborators representing varied backgrounds, roles, and interests to be engaged in the process of identifying barriers to intervention implementation and strategies to address them early on [20, 21]. Further, GCM bases the selection of intervention components and implementation strategies on the experiences and preferences of participants and encourages equal participation by everyone with a vested interest [21–23]. The GCM process (Fig. 1) consisted of 5 steps: 1) Brainstorming; 2) Sorting; 3) Rating; 4) Approval of Labels; and 5) Intervention Feedback. The Brainstorming step, a qualitative method, simulates a focus group discussion. Participants are asked to respond to a single prompt developed by the research team. Participants generate unlimited, unique responses to the prompt. Responses are recorded and displayed in real time such that participants can see previously generated statements. The sorting step reinforces the participatory nature of GCM by engaging participants in organizing the brainstormed statements into categories based on their perceived conceptual similarities. This process parallels qualitative thematic analysis, as it relies on participants’ interpretations to identify meaningful patterns among the responses. The rating step quantifies the brainstormed responses based on a scale defined by the research team. For developing this intervention, we chose for participants to rate statements using a 5-point Likert scale based on importance and feasibility to help prioritize intervention components and implementation strategies. Statement ratings are relative judgments or values assigned by participants, further demonstrating the participatory approach of GCM. Approval of Labels and Intervention Feedback are also participatory and inclusive in interpreting the GCM results and guiding intervention development and implementation.

The Black CACTus is composed of hair stylists, hair salon clients who identify as Black women, healthcare professionals, and representatives of community organizations that focus on the health and wellness of Black communities. The Black CACTus was formed by KP to provide insight and guidance on community-relevant health issues and support in engaging the community to address health disparities. The study team has engaged with these members in developing health programming and conducting research in various capacities over several years. However, this was the first time they were convened to conduct group concept mapping; therefore, the feasibility of conducting concept mapping with the Black CACTus was also assessed. In our previous work to understand preferences and perspectives on salon-based health promotion, salon clients who identify as Black women stated they prefer that their stylist partner with a medical professional or health/scientific expert when participating in health promotion [15]. They also noted a higher level of influence from and comfort with discussing certain topics (e.g., reproductive, sexually transmitted infections, etc.) with hair stylists who are women and/or Black/African American [15]. Because this study is focused on HPV/Cervical Cancer screening, it was decided to recruit Black CACTus members who could satisfy these preferences and provide additional relevant insights on the intervention. Therefore, to develop a salon-based HPV self-sampling intervention, we recruited Black CACTus members who represent one of the following: 1) healthcare professionals who provide gynecological care, 2) hair stylists who identify as women, or 3) salon clients who identify as Black women. All participants provided informed consent, completed a brief demographics survey, and were compensated for their time spent on each activity. The University of Arizona Institutional Review Board approved all study materials and activities.

### Data collection

The first three steps (brainstorming, rating, and sorting) of the GCM process were conducted using the web-based platform, groupwisdom™, and the final two steps (labels approval and intervention feedback) were completed in REDCap [24, 25]. The groupwisdom™ web-based platform enables GCM to be conducted in a convenient, low-burden, accessible manner for project participants while facilitating data collection, management, and analysis for researchers. The platform can be used anytime from any location using any computing or smart mobile device with internet access. Data collection can be adapted for virtual and in-person methods, and instructions and messages are customizable for each project. Privacy settings can be set with anonymous or minimally identifiable participant information while allowing researchers to track participant engagement and progress. Participants were sent an email with a link to the corresponding platform for data collection at each step. The groupwisdom™ and REDCap platforms were populated with the study objective and instructions for each step [24, 25]. Participants were given one week to complete each step.

The Research Administrator performed a quality check of the data after each step and accepted/rejected the data accordingly. The Research Administrator contacted participants whose data were rejected to clarify instructions, answer questions, and provide the participants with a new link to complete the data collection step.

Step 1- Brainstorming: Participants generated statements in response to the prompt “To be successful, cervical cancer (HPV) self-sampling screening for Black women in the salon setting should include….” Participants were encouraged to enter as many statements as desired. A real-time, running list of statements from all participants was visible in the platform.

After the brainstorming step, two team members (KP and NM) reviewed the statements. Duplicate statements were deleted, and complex statements encompassing more than one strategy were divided into multiple statements. Grammatical errors were edited while maintaining the authentic voices of participants. A final list of statements was approved by consensus between KP and NM.

Step 2- Sorting: Participants sorted the statements generated during brainstorming into categories based on their interpretation of the statements. They created a label for each category that captured the collective meaning of the statements. Instructions were given to sort every statement and provide a label for every category. There was no limit placed on the number of categories created.

Step 3- Rating: Participants rated each statement on the importance of each strategy statement using a Likert scale ranging from 0 (not important at all) to 5 (extremely important) based on the impact each strategy would have on the implementation of the HPV self-sampling intervention. Then, they rated each strategy statement on its feasibility using the Likert scale from 0 (not feasible at all) to 5 (extremely feasible).

Step 4- Approval of Labels: A Cluster map with labeled categories and corresponding strategy statements for each category was presented to participants. Participants reviewed the labels and the list of corresponding statements for each cluster. Participants were asked to approve/disapprove labels and suggest new labels for categories as appropriate. They were asked to provide feedback on the inclusion of strategy statements into each category using open-ended text boxes in REDCap.

Step 5- Intervention Feedback: Participants reviewed and provided written open-ended feedback on a draft salon-based HPV self-sampling intervention in REDCap. Each section of the intervention was uploaded as a PDF file in REDCap where participants could read and review the details. Feedback was solicited for each component of the intervention so participants could focus their responses accordingly. Participants were asked if each element of the intervention best captured the strategies identified and prioritized from the concept mapping process. They were encouraged to make further recommendations, including additions and deletions to intervention components and implementation strategies.

### Data analysis

The groupwisdom™ software conducts multidimensional scaling and hierarchical cluster analysis to generate the concept maps.

To create the concept maps, the groupwisdom™ software used data from the sorting step to create a similarity matrix. In this matrix, a numerical value of similarity was assigned to any two strategy statements based on the number of participants who sorted them into the same category. Statements that were grouped together more frequently by participants are closer to each other on the map. Then, hierarchical cluster analysis is conducted by the software to determine where to apply boundaries around the groups of statements into thematically related “clusters.” Ratings for each statement and cluster are then averaged. Statements were then mapped on a 2-dimensional plane based on their relation to each other to develop the concept map and summarize ratings. Next, the most appropriate number of categories to include in the final concept map was decided by KP and NM in an iterative process. First, upper and lower cluster number limits were established. Then starting with the upper limit and moving down, merged statements were reviewed at each cluster level. The final cluster level was identified as having retained the most valuable detail between clusters while maintaining thematic relevance of merged clusters. Determining the number of clusters for the final concept map is a subjective, project-specific decision guided by the goals of the project, the context in which the results will be applied, and the intended audience for the findings.

The final cluster map was determined to have the best representation of the homogeneity of statements within clusters and the distinction between clusters. The research team reviewed and revised the cluster names to best reflect the themes of the statements in each cluster, considering the suggested category names given by participants during the sorting step.

## Results

We engaged 10 Black CACTus members in this project. The members included three medical professionals (one family practice, one internal medicine, one obstetrician/gynecologist), three hair stylists, and four hair salon clients. All members identified as women and Black/African American race. Two participants identified as more than one race: Black and Asian, Black and Latina, and Black and White. The average age of all participants was 38 years. Hair stylists and medical professionals reported being in their professions for an average of 11 and 16 years, respectively. Participant characteristics are shown in Table 1.

**Table 1:** Participant Characteristics (n = 10)

Characteristics	n	%
**Role**		
Stylist	3	30
Client	4	40
Medical Professional	3	30
**Race/Ethnicity **^**a**^		
Black/African American	10	100
White	1	10
Asian/Asian American	1	10
Pacific Islander	0	0
Native American	0	0
Latino/Latina/Latine/Hispanic	1	10
**Mean (SD)**		
Age (year)	40.91	(11.15)
**Time in Role (y)**		
Stylist	11	(6.93)
Client	12.25	(9.64)
Medical Professional	15.67	(8.62)

^a^Proportions in the table do not sum to 100% as respondents could choose all races with which they identify

**Fig. 2:**
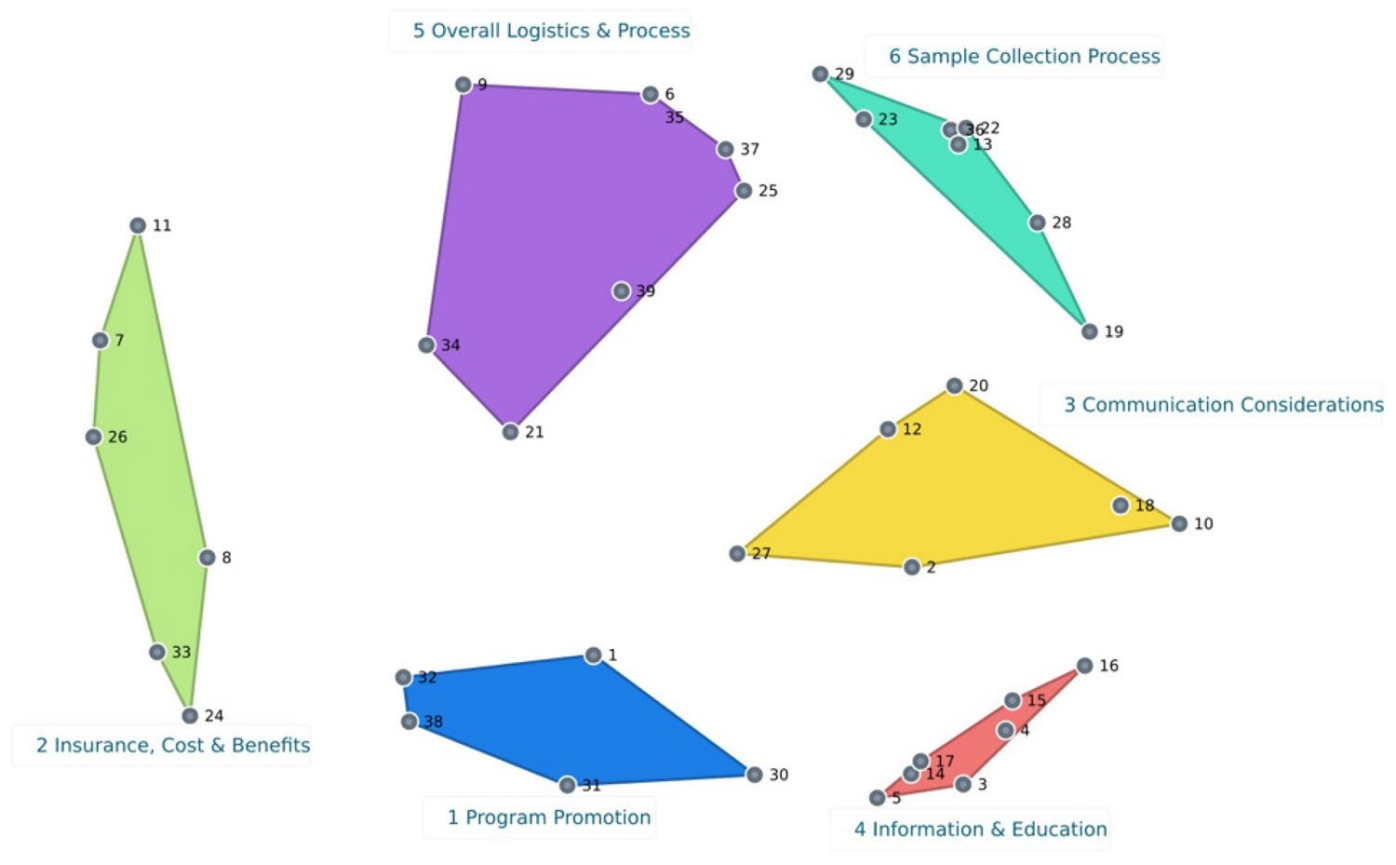
Final 6-cluster map

The brainstorming step yielded 38 statements. Further refinement of the statements and removal of duplicates by two members of the research team resulted in 39 statements. Participants created an average of five piles during sorting. A final Cluster Map of six clusters (Fig. 2) with a stress factor of 0.263 was chosen. The lower stress factor of 0.263 suggests a better overall fit and representation of the input data on the two-dimensional map [22]. By consensus of the research team and the study participants, the six clusters were labeled: 1) Program Promotion, 2) Insurance, Cost and Benefits, 3) Communication Considerations, 4) Information and Education, 5) Overall Logistics and Process, and 6) Sample Collection.

**Fig. 3:**
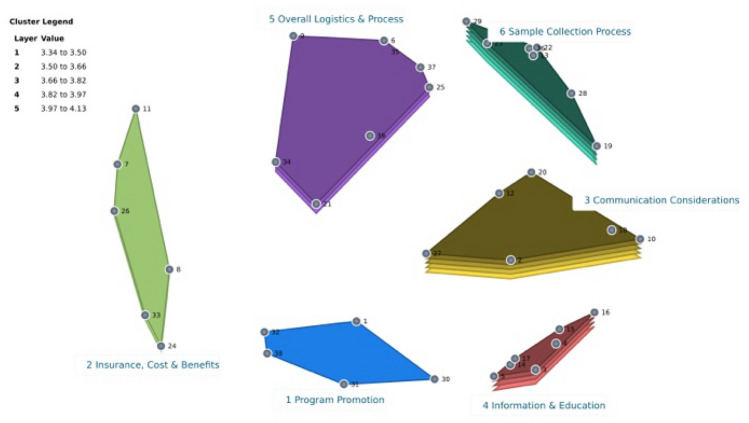
Importance cluster rating map

**Fig. 4:**
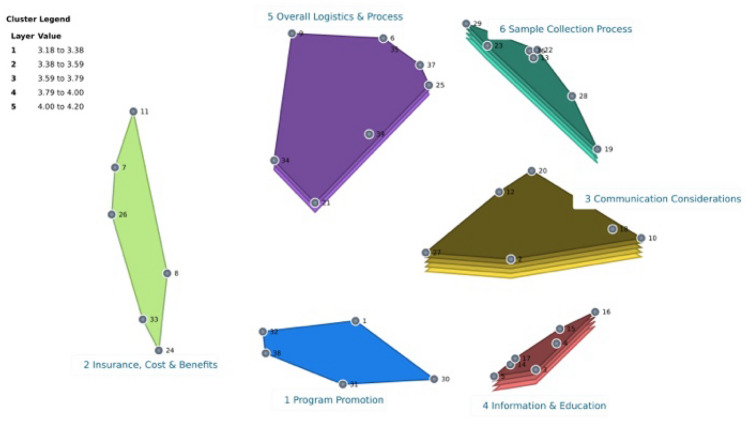
Feasibility cluster rating map

**Fig. 5:**
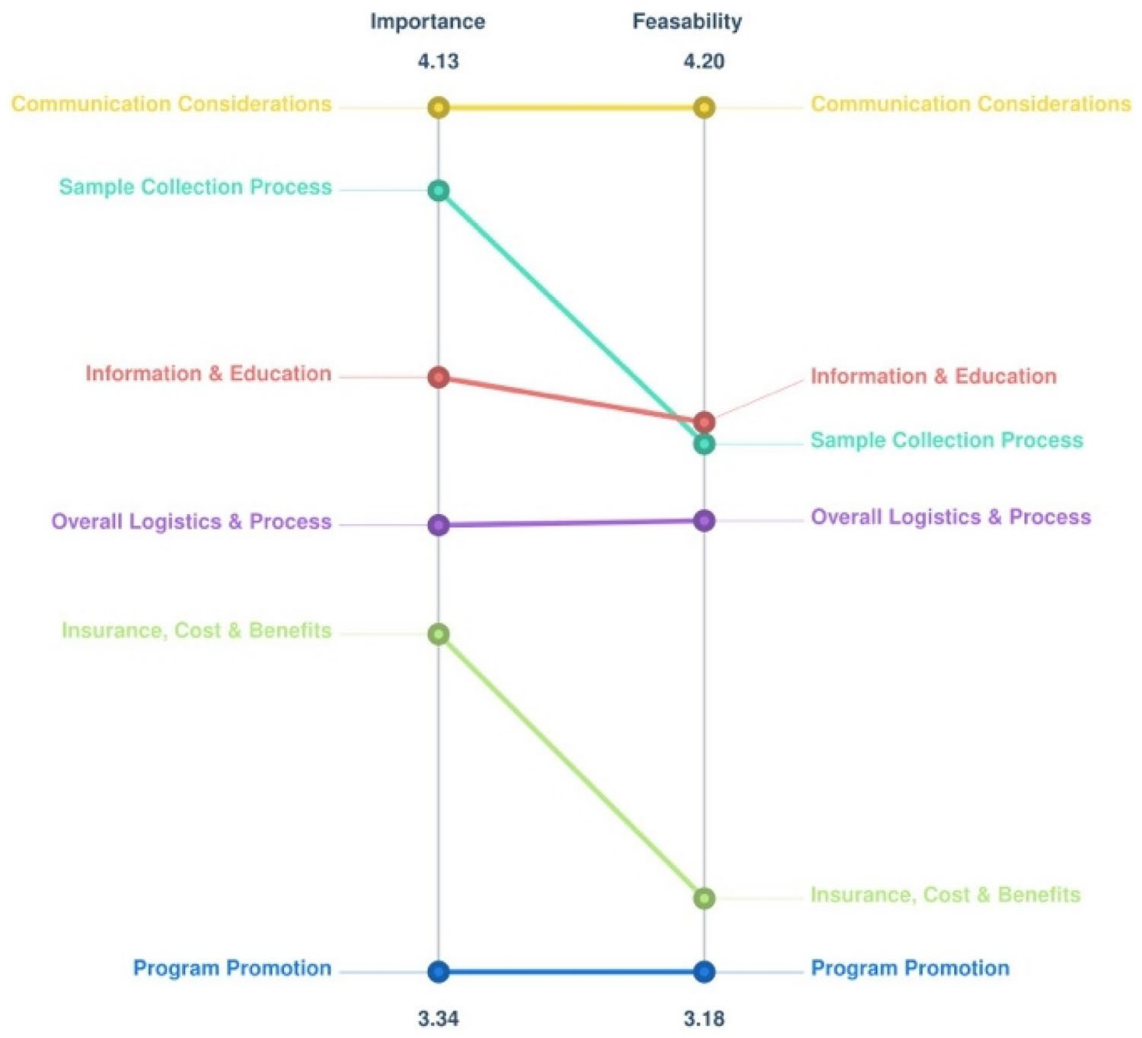
Pattern match of importance versus feasibility

Clusters (3) Communication Considerations and (6) Sample Collection had the highest average ratings for importance at 4.13 and 4.06, respectively. The Importance Cluster Rating Map (Fig. 3) illustrates the degree of importance for each cluster, with more layers representing a higher importance rating. The cluster rated the most feasible was “3: Communication considerations” with a rating of 4.20; this was followed by cluster “4: information & education” at 3.83 and “6: sample collection” at 3.80 (Fig. 4). The Pattern Match (Fig. 5) shows that when importance and feasibility were considered together, clusters 3, 4, and 6 were among the highest rated. Feedback from the label approval step revealed that most participants agreed with the 6-cluster map configuration, and on average, the labels for each cluster had a 92% approval rate (answered “Yes” to “Do you agree with the name of Cluster #”). Table 2 provides suggested revisions to cluster labels by participants. The study team maintained the cluster labels as presented, given the high approval rating and similarity to any suggested revisions. There was general “reservation” about including Statement 21, “attractive packaging” in Cluster 5 by the study team and participants. It was decided to keep Statement 21 in Cluster 5 to maintain the 6-cluster map as approved by consensus and to preserve the lower discrepancy between the input matrix data and the distances of those data on the map, as indicated by the stress factor.

**Table 2:** Suggested Cluster Labels

Cluster	Research team suggested Cluster name	Participant suggested cluster name
1	Program promotion	Program marketing
2	Insurance, COST, and Benefits	Affordable and Beneficial
3	Communication Considerations	*No Suggestions*
4	Information and education	*No Suggestions*
5	Overall logistics and process	Additional benefits; overall logistics and directions; overall logistics and instructions
6	Sample collection process	Simple collection process; sample collection directions; sample collection instructions

A draft salon-based cervical cancer screening and prevention program was developed based on rating data. Considering statements from the top-rated clusters, Communication Considerations, Information and Education, and Sample Collection Process, there was overlap of themes that emerged. There was overwhelming preference for clear, culturally relevant, and thorough education and communication. This included education about HPV, cervical cancer (risks, prevention, testing options, and treatment) and communication spanning from HPV self-collection instructions to being informed on testing results and follow-up care. Participants felt it was important that stylists receive education and training about HPV, cervical cancer, and self-collection screening to facilitate intervention implementation and be a trusted resource to guide clients throughout the process. From the Sample and Collection Cluster, participants highly ranked having clean, private spaces for self-collection. The draft program, prioritized by these clusters and statements rated as most important and feasible, resulted in three components, “Development of Culturally Appropriate Educational Materials, Training for Hair Stylists, and the salon-based HPV self-collection intervention.” Overall, participants felt the draft program addressed the factors and themes identified in the GCM process. Participants’ feedback will be used to further refine the program components to be piloted in a future study.

## Discussion

Community-based participatory research has been credited with addressing health inequities by engaging local communities in acknowledging the unique contexts of each community and setting [26]. Group concept mapping (GCM) is a useful and rigorous methodology for collaborating with community members to develop community-based health interventions [27]. To our knowledge, this is the first study to include the perspectives of hairstylists, salon clients, and medical professionals in a group concept mapping process to inform a salon-based HPV self-collection intervention. Using the groupwisdom™ software allowed our Black CACTus members to participate for the first time in GCM in a convenient and accessible format. As a result, a 6-cluster map was generated that includes six areas of consideration for salon-based cervical cancer screening: Communication Considerations, Sample Collection Process, Information and Education, Overall Logistics and Process, Insurance Cost and Benefits, and Program Promotion were the concepts identified by participants to be prioritized and incorporated into a salon-based HPV self-collection intervention.

Gathering the diverse opinions of the Black CACTus members, representing stylists, salon clients who identify as Black women, and medical professionals, offers broader contextual and cultural considerations for a salon-based HPV self-collection intervention. The intervention developed is strengthened by the consensus of the Black CACTus on the importance and feasibility of “communication considerations,” the “sample collection process,” and “information and education.” These identified concepts and considerations align with the literature that reports a need for better communication and education regarding cervical cancer risk and prevention and screening options and guidelines [28–31]. Participants emphasized the importance of tailoring educational efforts to resonate with Black women and the hair salon setting while also overcoming communication barriers by offering straightforward, reliable information. Other studies have documented Black women’s attitudes and beliefs about HPV self-collection, reporting a lower sense of self-efficacy to perform the test but a willingness and preference for self-sampling [30, 32–34]. This lack of confidence is due to a perceived lack of knowledge and skills to participate in HPV self-collection [35]. Our results suggest developing clear, effective educational materials and self-sampling instructions can address these concerns among Black women who are amenable to trying self-sampling.

By engaging Black CACTus members in this group concept mapping process, we facilitated joint meaning and consensus to inform the development of a salon-based HPV self-collection intervention. Engagement with the Black CACTus members demonstrates leveraging community assets through the inclusion of vested community members in the formative phase of an intervention, thereby informing important intervention targets, increasing the potential for intervention relevance, and acceptability for the priority population. Concept mapping enabled community members to operationalize various factors to be considered for the successful implementation of a salon-based HPV self-collection intervention within their communities. The results from our study suggest the importance of communication considerations and information and education. As such, these recommendations are considered for intervention development.

Efforts are underway in the U.S. to evaluate the usability and acceptability of HPV self-collection in clinical settings. This nationwide, multi-site clinical trial aims to increase access for high-burdened and underserved populations. However, healthcare innovations often fail to reach those most in need due to a lack of a health equity lens in implementation planning [36]. It is crucial to use culturally tailored strategies and consider the historical and current contexts in which Black women engage with the healthcare system and medical research to avoid perpetuating existing disparities [37–39]. Direct-to-consumer self-testing is cost-effective and convenient and has been instrumental in increasing access to medical testing and empowering patients to be proactively engaged in managing their health [40–42]. Community settings, like hair salons, should be considered to expand the accessibility of HPV self-collection testing. Although healthcare providers remain essential to cervical cancer screening efforts, respected community figures—like hair stylists—can also play a significant role by extending support as community health workers, health ambassadors, or lay health advisors to enhance communication and education about HPV self-collection for cervical cancer screening.

### Strengths and limitations

A key strength of this study is that we used the group concept mapping method to garner input from vested community members, including hair stylists, salon clients, and medical professionals with differing perspectives and expertise, as a CPBR approach to understand the challenges and facilitators of a hair salon-based HPV self-sampling intervention for Black women. A potential limitation of the study is using online software for information gathering during the GCM process. This may have restricted access to additional insights and non-verbal communication cues gained from in-person group concept mapping, particularly to understand feedback on the proposed intervention. However, we used open-ended questions to elicit information from participants, whereby they were able to provide unrestricted feedback on all components of the intervention. The online platform also proved to be low-burden and allowed for participation from community members at their convenience. Data from the groupwisdom™ system showed participants often accessed the system during late evening hours and oftentimes completed each step of the GCM process over the course of a few days. Conducting GCM in a remote environment also provided a level of anonymity that potentially reduces social desirability bias that can arise during in-person group-based qualitative data collection methods.

## Conclusions

This study provides a foundation for the development of a salon-based HPV self-sampling intervention to increase cervical cancer screening among Black women through group concept mapping with vested community members. It is important to develop effective evidence-based interventions addressing multilevel barriers and in friendly environments (safe spaces) to reduce cervical cancer health disparities. HPV self-sampling has been conducted in various settings nationally and globally. To our knowledge, HPV self-sampling has yet to be tested in a salon-based setting in the U.S. Hence, it was essential to obtain information on potential barriers and facilitators for the development of a tailored cervical cancer screening intervention for Black women in the hair salon setting. Culturally tailored interventions can potentially increase the likelihood of positive intervention effects among racial/ethnic minority populations [43]. Further, contextually informed strategies can mitigate implementation challenges often seen in non-traditional, “real world,” and community-based settings. The findings of this study will inform the development of a culturally and contextually appropriate HPV self-sampling intervention to be delivered by trained hairstylists who provide services for Black women and are trusted and respected in Black communities.

## Data Availability

The datasets used and/or analyzed during the current study are available from the corresponding author upon reasonable request.
